# Fabrication of Random Microlens Array for Laser Beam Homogenization with High Efficiency

**DOI:** 10.3390/mi11030338

**Published:** 2020-03-24

**Authors:** Li Xue, Yingfei Pang, Wenjing Liu, Liwei Liu, Hui Pang, Axiu Cao, Lifang Shi, Yongqi Fu, Qiling Deng

**Affiliations:** 1 University of Electronic Science and Technology of China, Chengdu 610054, China; xueli2553@163.com (L.X.); lwjoptics@163.com (W.L.); 2 Institute of Optics and Electronics, Chinese Academy of Sciences, Chengdu 610209, China; yfpang7647@163.com (Y.P.); liuliweineko@163.com (L.L.); ph@ioe.ac.cn (H.P.); dengqiling@ioe.ac.cn (Q.D.)

**Keywords:** chemical etching, random microlens array, beam homogenization, laser

## Abstract

The miniaturized and integrated microlens array (MLA) can effectively achieve the beam homogenization, compactness and miniaturization of laser systems. When the high-coherence laser beam is homogenized by means of using the MLA, interference fringes will occur in the homogenized light spot due to the periodicity of the MLA, which seriously affects the uniformity of the homogenized light spot. To solve this problem, a novel random microlens array (rMLA) structure was proposed for the purpose of achieving beam homogenization. The coherence in the homogenization process is suppressed by means of breaking the periodicity of the MLA. The homogenized light spot with a high energy utilization is then obtained accordingly. In the fabrication process, a clever method of combining chemical etching with lithography technology is performed to fabricate a honeycomb rMLA and a rectangular rMLA. The experimental results show that the energy utilization rate of the two types of the rMLAs is about 90%, and the uniformity of the homogenized light spots generated by the honeycomb rMLA and the rectangular rMLA are more than 80% and 85%, respectively. Meanwhile, fully cost-effective fabrication is possible to be realized.

## 1. Introduction

Lasers have been widely applied in many fields, such as medical treatment [1,2], laser illumination [3,4], laser detection [5,6] and satellite communication [7,8]. However, the intensity distribution of a laser is usually in a Gaussian profile, which is always required to be converted into a flat-top profile so as to achieve the desired effect, e.g., laser welding [9,10], an optical system of exposure machine [11], laser drilling [12,13] and laser projection [14,15]. There are some methods to achieve beam homogenization, such as an aspheric lens group [16,17,18], free-form lens [19,20,21], diffractive optical element (DOE) [22,23,24] and microlens array (MLA) [25,26,27].

For the aspheric lens group and free-form surface lens, the former has a high light energy utilization rate and high beam uniformity, which can be applied to high-power lasers, while the latter is a simple optical system with high design freedom which can be used to effectively achieve high uniformity. However, the above two methods require nanometer surface roughness, which increases the fabrication difficulty. In addition, because of the limitation of the sizes of the corresponding lenses, it is a challenge to realize the miniaturization and integration of the system. The DOE can be used to achieve the miniaturization and integration of the systems, and also has high design freedom. Nevertheless, the DOE is generally only suitable for a single wavelength, and cannot be used to shape the beam in a broad spectral range. Meanwhile, energy efficiency depends on the number of phase levels of the DOE; the greater the number of phase levels is, the higher the energy efficiency will be, but also the more complex the fabrication. In contrast, the MLA is widely applied to the field of multi-wavelength laser beam homogenization to achieve the miniaturization and integration of the systems, due to its merits of a high energy utilization rate, extremely simple structure and high beam uniformity.

Many related research works of beam homogenization by means of using MLA have been reported before. In 2017, Hang et al. [28] used a monolithic periodic MLA to homogenize the laser beam. The collimated laser beam is subdivided into multiple beamlets by the MLA, and then the beamlets are superposed and recombined with each other to suppress the nonuniformity by a focusing lens. The uniformity of the obtained homogenized light spot is 91.1%. The energy utilization rate of the MLA with anti-reflection coating can reach 93%. In order to homogenize the incident laser beam with a certain divergence angle, a method on the basis of two periodic MLAs is proposed by Hwang et al. [29] to obtain a homogenized light spot with high energy efficiency in 2017. However, when high-coherence lasers are shaped by their method, interference between beamlets will also occur due to the periodicity of the MLA, the resulting interference fringe appears in the obtained homogenized light spot and the uniformity of the homogenized light spot will be deteriorated. In order to eliminate the effect of interference on the homogenized light spot, Xianzi et al. [30] prepared a 16-level random phase fresnel lens array by means of setting the phase of sub lenses of the fresnel lens array to a random arrangement in 2019. This broke the coherence between the beamlets, and obtained a homogenized light spot with a uniformity of 83%. However, the fabrication of the 16-level random microlens array (rMLA) is complex. In 2019, Liu et al. [31] designed a continuous profile MLA with sub lenses of the random apertures and arrangements. It realized a breakthrough of the coherence of beamlets so that the uniformity obtained by simulation is able to reach 94.33%. However, they proposed to fabricate the rMLA by 3D printing technology, which is not yet mature.

At present, the fabrication technology of continuous rMLA is not yet mature. In contrast, we innovatively combined the traditional lithography exposure technology with chemical etching technology to propose a novel method to fabricate rMLA, which can be used to fabricate high-efficiency, low-cost and large-area rMLA. Meanwhile, in order to further reduce the surface roughness of microlenses, three kinds of chemical solutions were prepared in this paper: the fundamental chemical etching solution (deionized water, hydrofluoric acid solution (HF solution) (40%) and ammonium fluoride particles (NH_4_F)); the H_2_SO_4_ chemical etching solution (deionized water, HF solution (40%) and the H_2_SO_4_ (95–98%)); and the HNO_3_ chemical etching solution (deionized water, HF solution (40%) and the HNO_3_ (65–68%)), respectively. We compared the etching effects of the three kinds of solutions and obtained a best suitable solution ratio. Adopting the proposed method, we fabricated two types of rMLAs, which were the honeycomb rMLA and the rectangular rMLA, respectively. Moreover, a chromium film layer was plated on the surface of the glass substrate, which masterly avoided side etching and the curl-up of the edge. In addition, the high-quality surface of the microlenses can effectively guarantee the homogenization quality of the beam, and the randomness of the rMLA is able to successfully break the coherence of the laser beam so that the uniformity of the modulated beam can be improved. Section 2 describes the beam homogenization model of rMLA, while Section 3 demonstrates the fabrication process of the rMLA in detail, Section 4 shows experimental results and verifies the validity of our method and Section 5 is a summary.

## 2. Beam Homogenization Model of rMLA

**Figure 1:**
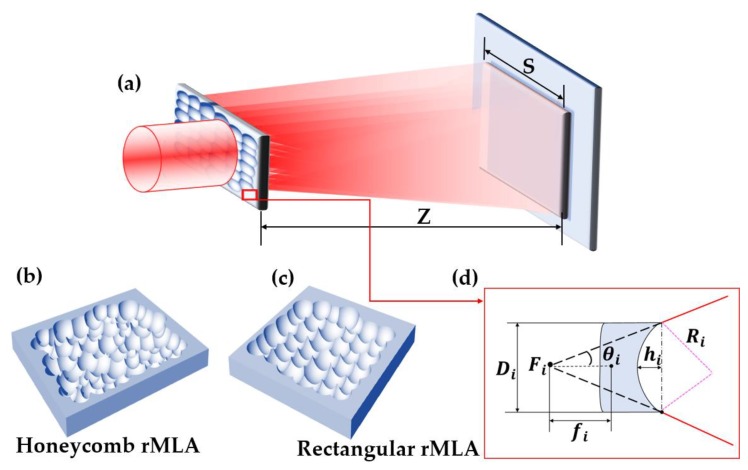
(**a**) Schematic diagram of random microlens array (rMLA); (**b**) structural parameters of microlens unit; (**c**) honeycomb rMLA; (**d**) rectangular rMLA.

The working principle of beam homogenization based on the random microlens array (rMLA) is shown in Figure 1a. The condition of interference among the beamlets is broken, and the influence of interference on beam homogenization is suppressed by the rMLA, as the phases of the beamlets are randomly distributed due to the random apertures, focal lengths and arrangements. According to this principle, the preparation of the two types of rMLAs have been proposed in this paper: one is the honeycomb rMLA, as shown in Figure 1b, which can be used to produce a circular homogenized light spot; and the other is the rectangular rMLA, as shown in Figure 1c, which can be used to produce a rectangular homogenized light spot. The structural parameters of sub lenses of the two types of rMLAs are shown in Figure 1d, including aperture *D_i_* and sag height *h_i_*. As can be seen from Figure 1d, the geometric relationship between the radius of curvature *R_i_* and the sag height *h_i_* can be obtained, expressed by Equation (1). According to the relationship between the radius of curvature and the focal length, the focal length ($f_{i}$) can be calculated by Equation (2), where n is the refractive index. Therefore, the divergence angle of the beam passing through the sub lenses can be calculated by Equation (3). Meanwhile, the divergence angle *θ_i_* of the entire light beam after passing through the rMLA is formed by the superposition of randomly distributed sub lenses, and is determined by the divergence angle of the sub lenses with a higher frequency of occurrence.

(1) $$R_{i}=\frac{D_{i}^{2}+4h_{i}^{2}}{8h_{i}}$$

(2) $$f_{i}=-\frac{R_{i}}{n-1}=-\frac{D_{i}^{2}+4h_{i}^{2}}{8(n-1)h_{i}}$$

(3) $$\theta_{i}={tan}^{-1}\frac{D_{i}}{2|f_{i}|}$$

## 3. Fabrication of Random Microlens Array (rMLA)

Fabrication of the rMLA is realized by the combination of chemical etching and lithography technology. Before the glass substrate is chemically etched, the chemical etching solution needs to be prepared and the substrate needs to be pretreated. The composition and proportion of the solution are super important, which will significantly affect the chemical etching rate and the profile of the rMLA in the experiment, and further affect the beam homogeneity as well. For substrate pretreatment, the surface of the substrate is necessary to be coated with chromium film as the masking layer of chemical etching. In particular, the fabrication of the rMLA requires that the random micropores array is processed on the surface of the chromium film, so that the chemical etching solution is able to react with the glass substrate.

### 3.1. Preparation of Chemical Etching Solution

In the experiment, ultra-white glass was selected as the substrate material, which has a transmittance of 91.5% in the visible regime. The main component of the selected substrate is SiO_2_, with a content of 73%. The contained impurities mainly involve Al_2_O_3_, Fe_2_O_3_, CaO, MgO, Na_2_O and K_2_O. Among them, CaO and Na_2_O occupy a relatively high proportion, which are 11% and 15%, respectively. The other impurities are low, at about 0.15%, which were not considered as key factors in this experiment. 

During chemical etching, three different chemical etching solutions were fabricated. The compositions of the chemical etching solution included deionized water, hydrofluoric acid solution (HF solution) (40%) and ammonium fluoride particles (NH_4_F), which was called the fundamental chemical etching solution. The proportion of ingredients was 10:3:1. Meanwhile, the units of deionized water and HF solution were “mL”, while the unit of NH_4_F was “g”. The HF solution is able to react with various oxides of ultra-white glass [32], and the chemical reaction formulas are expressed as Equations (4)–(6)

(4) $${\mathrm{SiO}}_{2}+4\mathrm{HF}\to {\mathrm{SiF}}_{4}+2H_{2}O$$

(5) $$\mathrm{CaO}+2\mathrm{HF}\to {\mathrm{CaF}}_{2}+H_{2}O$$

(6) $${\mathrm{Na}}_{2}O+2\mathrm{HF}\to 2\mathrm{NaF}+H_{2}O$$

where SiF_4_ will continue to react with the generated fluoride, which can be expressed by Equations (7) and (8). Among these, the reaction rate of CaF_2_ is slow and will be residues

(7) $${\mathrm{SiF}}_{4}+{\mathrm{CaF}}_{2}\to {\mathrm{CaSiF}}_{6}$$

(8) $${\mathrm{SiF}}_{4}+2\mathrm{NaF}\to {\mathrm{Na}}_{2}{\mathrm{SiF}}_{6}$$

where SiF_4_ will react with the NH_4_F added in the chemical etching solution, expressed as Equation (9), to accelerate the etching rate of the glass.

(9) $${\mathrm{SiF}}_{4}+2{\mathrm{NH}}_{4}F\to {\left({\mathrm{NH}}_{4}\right)}_{2}{\mathrm{SiF}}_{6}$$

**Figure 2:**
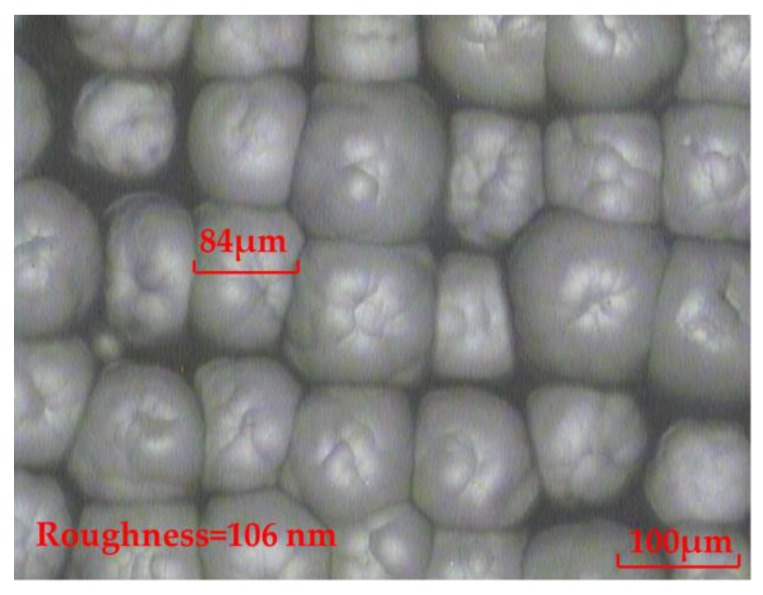
The rMLA fabricated by the fundamental chemical etching solution.

In the process of a reaction between the etching solution and glass substrate, the generated impurities CaF_2_ and Na_2_SiF_6_ with low solubility would adhere to the surface of the glass and prevent further etching. Although the chemical etching solution was continuously oscillated during the experiment to prevent impurities from adhering to the glass surface, the surface of the fabricated rMLA is not smooth, with a roughness of 106 nm, as shown in Figure 2. Thereby, a lot of stray light is generated during the beam homogenization process, which seriously affects the uniformity of the homogenized light spot. 

It was necessary to consider adding other chemical reagents to react with the generated impurities of the fundamental chemical etching to dissolve it in the etching solution. In this paper, the concentrated sulfuric acid (H_2_SO_4_) was selected to prepare the H_2_SO_4_ chemical etching solution. The components of the H_2_SO_4_ chemical etching solution were deionized water, HF solution (40%), and H_2_SO_4_ (95–98%). The proportion of ingredients was 5:2:2, and their units were “mL”. The diluted H_2_SO_4_ is able to react with the generated fluoride, which can be expressed as Equation (10):

(10) $$H_{2}{\mathrm{SO}}_{4}+{\mathrm{CaF}}_{2}\to {\mathrm{CaSO}}_{4}+2\mathrm{HF}$$

**Figure 3:**
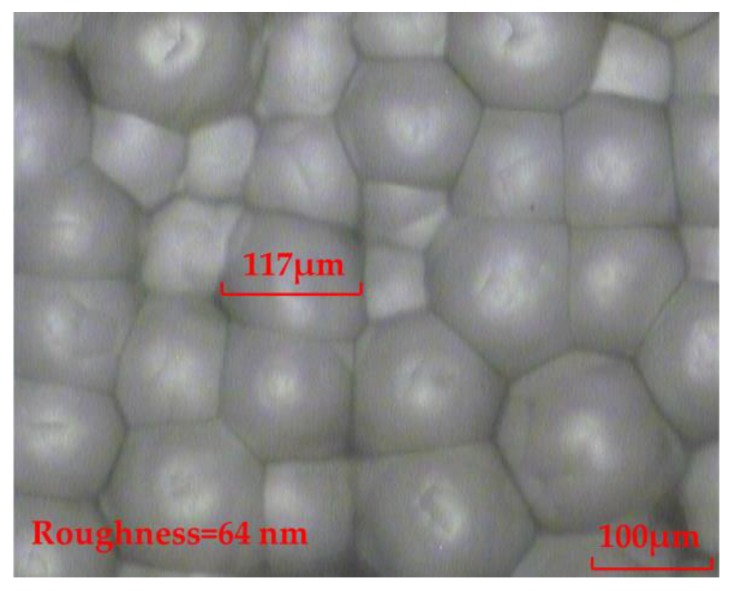
The rMLA fabricated by the H_2_SO_4_ chemical etching solution.

As can be seen from Equation (10), H_2_SO_4_ is able to react with CaF_2_. The produced CaSO_4_ will dissolve to some extent, but the remaining impurities still adhered to the glass surface. Although the chemical etching solution was also oscillated in the experiment, the impurities still hindered any further etching of the glass. The experimental result shows that the surface of the fabricated rMLA is improved compared to the fundamental chemical etching solution. However, the surface of the fabricated rMLA by the H_2_SO_4_ chemical etching solution is still not smooth, with a roughness of 64 nm, as shown in Figure 3, which will affect the uniformity of the homogenized light spot.

Based on the above analysis, we added the concentrated nitric acid (HNO_3_) to prepare the HNO_3_ chemical etching solution in order to improve the surface quality of the fabricated rMLA. The compositions of the etching solution were deionized water, HF solution (40%) and the HNO_3_ (65–68%). The proportion of ingredients was 5:2:2 and their units were “mL”. The diluted HNO_3_ is able to react with the generated fluoride, which can be expressed as Equation (11):

(11) $${\mathrm{CaF}}_{2}+2{\mathrm{HNO}}_{3}\to 2\mathrm{HF}+\mathrm{Ca}{\left({\mathrm{NO}}_{3}\right)}_{2}$$

**Figure 4:**
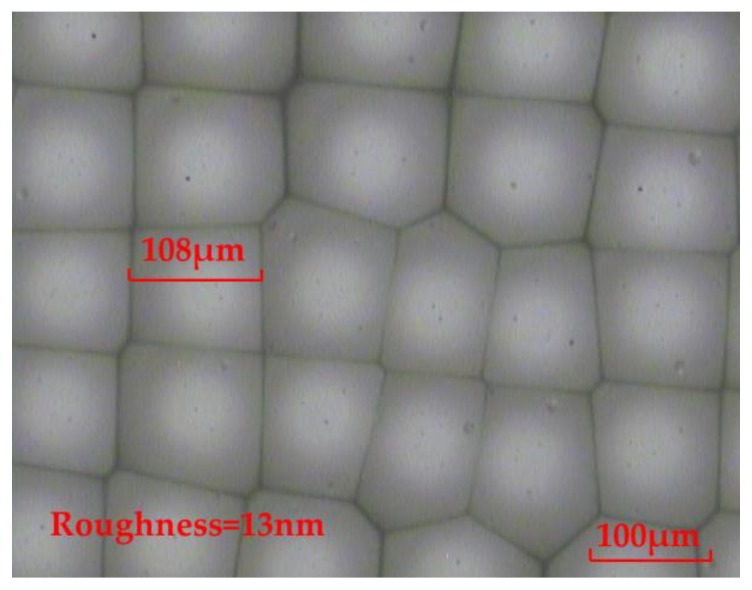
The rMLA fabricated by the HNO_3_ chemical etching solution.

From Equation (11), HNO_3_ is able to react with CaF_2_. Meanwhile, the generated Ca(NO_3_)_2_ is easily soluble in water. By continuously oscillating the chemical etching solution, the surface of the fabricated rMLA by the HNO_3_ chemical etching solution can be smooth, with a roughness of 13 nm, which reaches the surface quality of the optical device, as shown in Figure 4. Therefore, the HNO_3_ chemical etching solution is subsequently selected to carry out the experiment for fabricating the rMLA. 

### 3.2. Fabrication of rMLA

Two types of rMLAs are fabricated in this paper, which are the honeycomb rMLA and the rectangular rMLA, respectively. The fabrication requires the pretreatment of the surface of the glass substrate—that is, the random micropores array with different structures needs to be fabricated on the surface of the substrate. 

#### 3.2.1. Fabrication of Honeycomb rMLA

**Figure 5:**
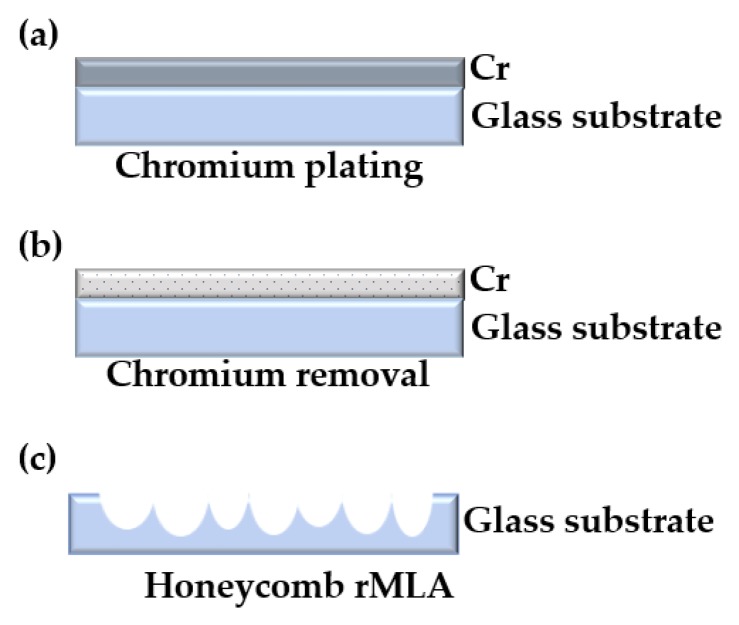
Fabrication schematic of honeycomb rMLA: (**a**) chromium plating; (**b**) corroded chromium film; and (**c**) honeycomb rMLA obtained by chemical etching.

**Figure 6:**
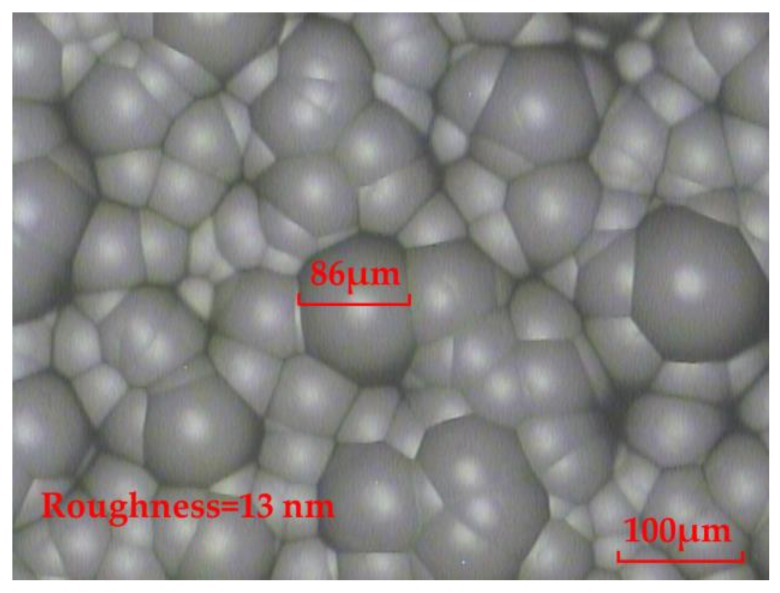
Microscopic image of the honeycomb rMLA.

For the fabrication of the honeycomb rMLA, a chromium film with a thickness of about 140 nm was first coated on a glass substrate, as shown in Figure 5a. Secondly, the glass substrate was put into the chromium removal solution and soaked for 10 s to etch a series of random micropores on the chromium film, as shown in Figure 5b. Finally, the preprocessed substrate was put into the chemical etching solution to fabricate the honeycomb rMLA. During the etching process, the etching solution was continuously oscillated and the substrate was cleaned every 10 min in order to prevent the detached chromium film from affecting the profile of the rMLA. Meanwhile, the total etching time was about 25 min. The experimental result observed by an OLYMPUS BX51 microscope is shown in Figure 6. As can be seen from the microscopic image of the fabricated honeycomb rMLA, the surface of the rMLA is composed of irregular microstructures, making the overall profile look like a honeycomb. Meanwhile, the surface of the honeycomb rMLA has quite a high filling ratio, which guarantees that the whole beam passing through the rMLA is able to be modulated, so that the uniformity of the generated light filed is further improved. Moreover, the surface of the honeycomb rMLA is extremely smooth, with a roughness of 13 nm, as shown in Figure 6, which verifies that high-quality rMLA can be fabricated by our method. However, due to the irregularity of the structure parameters of the honeycomb rMLA, it is difficult to measure the sagittal heights and the apertures.

#### 3.2.2. Fabrication of Rectangular rMLA

The fabrication of the rectangular rMLA, which is slightly different from the honeycomb rMLA, requires a mask predesigned and prefabricated. Then, through exposure, development, chromium removal and other processes, the random microporous structure on the mask plate was transferred to the chromium film of the substrate. Finally, chemical etching was performed to generate rMLA. 

**Figure 7:**
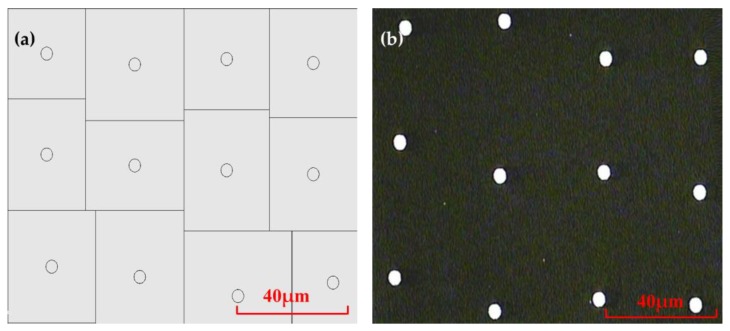
Mask part pattern: (**a**) rectangular randomly arranged mask data; and (**b**) microscopy pattern of mask made by laser direct writing technology.

The designed mask is a micropores array of random rectangular arrangement, as shown in Figure 7a. The radiuses of micropores are the same as 1.8 μm, and the distances between adjacent micropores varies randomly from 24 μm to 45 μm. The mask was fabricated by high-precision laser direct writing technology with a fabrication accuracy of ±250 nm. The pattern of the mask plate observed under the microscope is shown in Figure 7b.

**Figure 8:**
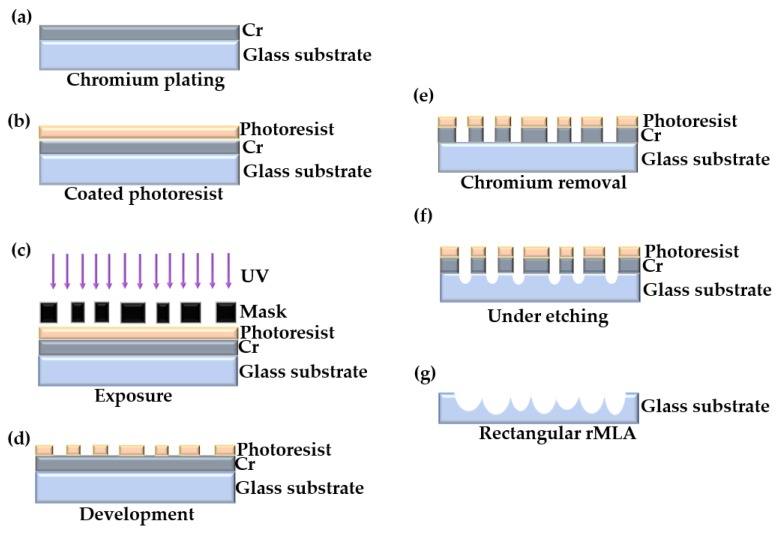
Fabrication schematic of rectangular rMLA: (**a**) chromium plating; (**b**) coated photoresist; (**c**) exposure; (**d**) development; (**e**) chromium removal; (**f**) under etching; and (**g**) rectangular rMLA obtained by chemical etching.

**Figure 9:**
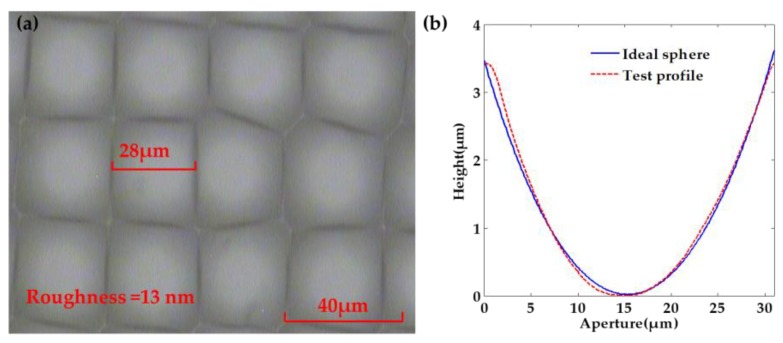
Micrographs and simulation profiles: (**a**) microscopic image of rectangular rMLA; and (**b**) comparison of microlenses profile and ideal sphere.

Subsequently, the chromium film, with a thickness of about 140 nm, was coated on the glass substrate, as shown in Figure 8a. The photoresist of the type AZ MIR-703 Photoresist (14 cp) (AZ Electronic materials, Somerville, MA, USA) was spin-coated on the glass substrate with chromium film at a speed of 4000 r/min with a time of 30 s. The thickness of the photoresist was about 700 nm, as shown in Figure 8b. The photoresist was then exposed by the exposure machine with a center wavelength of 365 nm. The exposure method was contact exposure with an exposure time of 20 s and the exposure power of 3 mW, as shown in Figure 8c. By the development with developer of AZ 300MIF (AZ Electronic materials, Somerville, MA, USA) for 35 s, the microporous structure on the mask was transferred to the photoresist, as shown in Figure 8d. The substrate was then put into the chromium removal solution for 40 s to transfer the microporous structure on the photoresist to the chromium film to complete the substrate pretreatment, as shown in Figure 8e. The substrate was finally put into the etching solution for etching. The etching solution penetrated from the micropores and contacted with the glass surface to produce a reaction. After the glass substrate was etched for a period of time, micropits were generated on the substrate surface, as shown in Figure 8f. The operation steps in the etching process were consistent with the fabrication of the honeycomb rMLA. During the etching process, the etching solution was continuously vibrated to prevent the falling chromium film from affecting the surface shape of rMLA. The glass substrate was cleaned every 10 min or so. After etching for about 40 min, the rMLA was obtained, as shown in Figure 8g. The experimental result obtained with the microscope is shown in Figure 9a, where it can be clearly seen that the structure is random due to the irregularity of arrangement of microlenses, the surface is smooth with a roughness of 13 nm, the filling ratio is high and the arrangement of rectangular rMLA is basically consistent with the arrangement of Figure 7b. Comparing the profiles data of the microlenses measured with the step profilometer (Stylus Profiler System, Dektak XT, Bruker, Karlsruhe, Germany) with an ideal spherical surface, it can be seen that the profile of the microlenses is almost spherical, and the fitting graph is shown in Figure 9b.

## 4. Experiments

**Figure 10:**
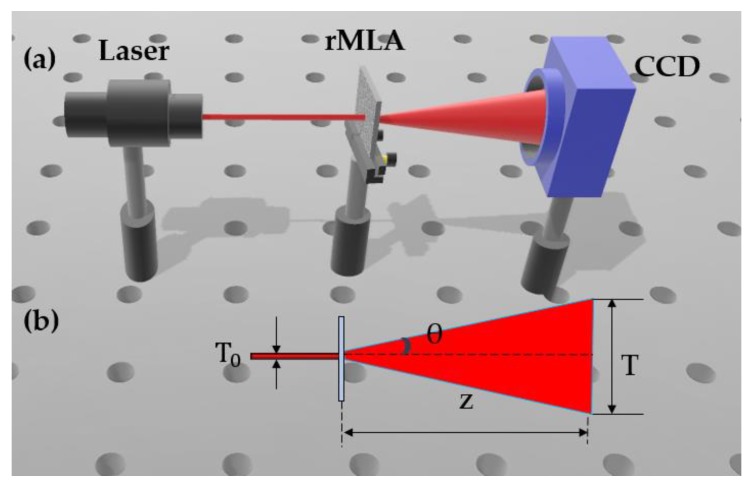
Experimental measurement: (**a**) the experimental setup; and (**b**) the diagram of geometric calculation method.

Based on the fabricated honeycomb random microlens array (rMLA) and rectangular rMLA, an optical setup was constructed to test the uniformity of the homogenized light spots generated by the two types of rMLAs, as shown in Figure 10a.

The rMLA was illuminated by a laser beam with a wavelength of 650 nm and diameter of 5 mm. After the incident light was modulated by the rMLA, a homogenized light spot was able to be obtained. The charges coupling device (CCD) was then used to collect and record the intensity of the homogenized light spot, as shown in Figure 10a. In order to characterize the divergence angle of the reconstructed light spot, the sagittal heights and the apertures of the fabricated rMLA needed to be measured. Subsequently, the divergence angle could be calculated by Equations (1)–(3). Using the theoretical equations, the divergence angle of the rectangular rMLA could be obtained. However, it was difficult to measure the sagittal heights and the apertures due to the irregularity of the structure parameters of the honeycomb rMLA, resulting in the divergence angle of the honeycomb rMLA not being directly calculated by the theoretical Equations (1)–(3). Therefore, the geometric calculation method was performed to calculated the divergence angle of the honeycomb rMLA, which would be described as follows. As could be seen from Figure 10b, the propagation distance *Z*, the size of the incident light $T_{0}$ and the reconstructed light spot *T* were able to be measured. Therefore, the geometric relationship of these four parameters (*θ*, $T_{0}$, *T* and *Z*) can be expressed as Equation (12).

(12) $$\theta =tan^{-1}\frac{T-T_{0}}{2Z}$$

The measured *T_0_* was 5 mm and *Z* was 27 mm, the homogenized light spot size *T* was calculated based on the MATLAB (version 7.1, MathWorks, Natick, MA, USA) numerical analysis software. At the edge of the spot, the dimension when the energy drops to 1/*e^2^* is recorded as *T*, which can be obtained by multiplying the number of pixels occupied by the light spot and the pixel size of the CCD. For the honeycomb rMLA, the number of pixels occupied by a circular light spot was 1177 at the central cross section, and the pixel size of the CCD was 7.4 μm. Therefore, the calculated *T* was about 8.7 mm. On the basis of the geometric calculation method, according to Equation (12), the divergence angle of the circular light spot could be obtained, which was about 7.8°. For the rectangular rMLA, the aperture and depth of sub lenses in multiple rMLA were measured by the step profilometer (Stylus Profiler System, Dektak XT, Bruker, Karlsruhe, Germany), according to Equations (1)–(3), the average value of departure divergence was about 22°. Meanwhile, the geometric calculation method was also performed to calculated the divergence angle of the rectangular rMLA. According to the experimental measurement, the number of pixels occupied by the rectangular light spot was 2161 at the central cross section and the calculated *T* was 16 mm. Therefore, according to Equation (12), it could be calculated that the divergence angle of the rectangular light spot was about 23°, which is basically consistent with the average divergence angle calculated by the theoretical Equations (1)–(3).

**Figure 11:**
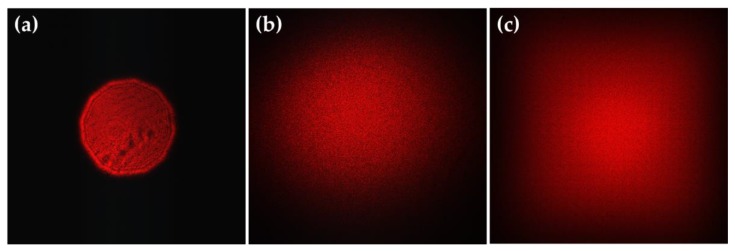
Homogenization spot effect collected by CCD: (**a**) the incident laser beam; (**b**) the circular homogenization spot reconstructed by the honeycomb rMLA; and (**c**) the rectangular homogenization spot reconstructed by the rectangular rMLA.

Subsequently, the uniformity and energy utilization of the homogenized light spot were tested. The incident light was an uneven light spot with a wavelength of 650 nm, as shown in Figure 11a. A circular homogenized light spot was generated by a honeycomb rMLA, as shown in Figure 11b, and a rectangular homogenized light spot was generated by a rectangular rMLA, as shown in Figure 11c. The uniformity of the light spot was calculated by using Equation (13), where the uniformities of the circular spot and the rectangle spot were about 81% and 88%, respectively. The power (*P_in_*) of the input beam measured with the power meter was about 3.5 mW, and the output powers (*P_out_*) after passing through the honeycomb rMLA and the rectangular rMLA were all about 3.2 mW. According to Equation (14), the energy utilization rate was about 90%. Therefore, it can be concluded that both the honeycomb rMLA and the rectangular rMLA have a quite high energy utilization rate.

(13) $$RMS=\sqrt{{\sum_{j}^{N}\left(I_{j}-\overset{-}{I}\right)}^{2}/N}$$

(14) $$P=\frac{P_{out}}{P_{in}}$$

**Figure 12:**
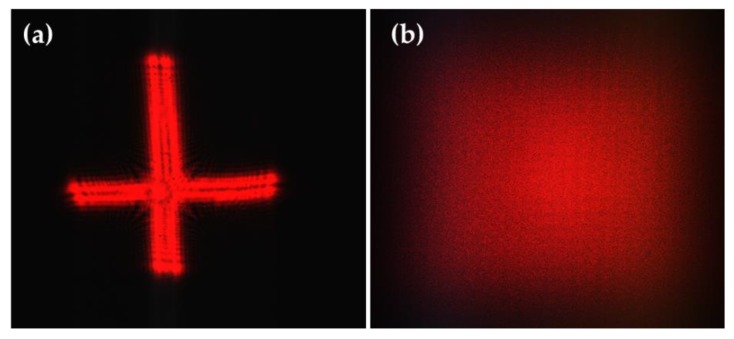
Beam homogenization with an arbitrary shape laser beam: (**a**) free-form incident laser beam; and (**b**) the reconstructed rectangular homogenization spot.

Many incident laser beams with arbitrary profiles and uneven intensity distribution, in addition to those mentioned above, can also be modulated by the rMLA to generate a homogenized light spot. For instance, utilizing the fabricated rectangular rMLA, the laser beam with the cross spot (Figure 12a) can be shaped into a rectangular homogenized light spot (Figure 12b).

## 5. Conclusions

This paper proposed a method of combining chemical etching with lithography technology to prepare the random microlens array (rMLA) to break the periodicity of the MLA, suppress the coherence during the homogenization process and obtain a homogenized light spot with high energy utilization. Through measurement, the uniformity of the circular light spot was about 81%, while the rectangular light spot was about 88%. The energy utilization rate of the homogenized light spots by the two types of rMLAs was about 90%. The designed rMLA can be expected to be used in laser welding machines, medical treatment, exposure machine lighting systems and other fields.
